# Modeling *Mycobacterium tuberculosis* pathogenesis in lung epithelial organoids reveals strain-specific host responses and intercellular crosstalk

**DOI:** 10.1016/j.jbc.2025.110534

**Published:** 2025-07-28

**Authors:** Ruiqi Zhang, Fusheng Yao, Yanhong Huang, Wenqi Liu, Shumin Liao, Junyan He, Siqi Li, Zhaoqin Wang, Liang Li, Guoliang Zhang

**Affiliations:** 1 National Clinical Research Center for Infectious Diseases, Shenzhen Third People's Hospital, Southern University of Science and Technology, Shenzhen, China; 2 School of Medicine, Southern University of Science and Technology, Shenzhen, China; 3 Joint Laboratory of Guangdong-Hong Kong Universities for Vascular Homeostasis and Diseases, Department of Pharmacology, School of Medicine, Southern University of Science and Technology, Shenzhen, China

**Keywords:** human alveolar epithelial organoids, *Mycobacterium tuberculosis*, coculture model, cell–cell interaction, ferroptosis

## Abstract

Emerging studies have identified alveolar epithelial cells as a conducive niche for *Mycobacterium tuberculosis* (Mtb) replication and spread during early infection. However, the host–pathogen interactions and intercellular crosstalk within the lung epithelial microenvironment remain inadequately understood. Here, we developed a lung epithelial organoid coculture model and exposed it to the virulent H37Rv strain or the avirulent *Bacillus* Calmette–Guérin (BCG) strain to investigate tuberculosis (TB) pathogenesis. Transcriptomic analyses revealed that Mtb infection markedly alters cell death patterns in organoids and modulates signal transduction pathways in peripheral blood mononuclear cells (PBMCs). Western blot indicates the H37Rv strain induced ferroptosis, autophagy, and apoptosis while suppressing necroptosis in organoids. In contrast, BCG predominantly enhanced autophagy. PBMCs also exhibited strain-specific responses, with BCG strongly activating the Hippo and Notch signaling pathways, whereas H37Rv primarily engaged the tumor necrosis factor (TNF) signaling pathway. Furthermore, Mtb significantly reshaped the paracrine and autocrine signaling dynamics between PBMCs and organoids. NicheNet network analysis identified TNFSF15 and brain-derived neurotrophic factor (BDNF), induced by H37Rv, as key mediators. Experimentally, overexpression of TNFSF15 and BDNF suggested that TNFSF15 from organoids promoted BDNF expression in PBMCs *via* paracrine signaling. In turn, BDNF from PBMCs then inhibited ferroptosis in organoids, contributing to restrict Mtb growth. Overall, our study provides a conceptual framework for understanding the mechanisms of TB pathogenesis within alveolar epithelial cells and offers valuable insights to prevent and control TB transmission in humans.

Tuberculosis (TB), a centuries-old infectious disease caused by *Mycobacterium tuberculosis* (Mtb), continues to pose a significant global health challenge, particularly in developing countries (1). Urgent efforts are needed to unravel the intricate mechanisms underlying Mtb infection to advance our understanding and treatment strategies.

Traditionally, macrophages have been recognized as the primary target cells for Mtb. However, recent discoveries have expanded this view to include lymphatic endothelial cells (2), hematopoietic stem cells (3), and notably alveolar epithelial cells (AECs) (4). Among these, AECs serve as a pivotal niche for bacterial recognition, uptake, replication, and dissemination during early infection stages (4, 5, 6). The interaction between Mtb and AECs significantly shapes host cellular responses by modulating gene expression and metabolic processes, which in turn affect intercellular communication within the lung microenvironment (7, 8). For instance, Mtb has been shown to induce pulmonary fibrosis *via* the transforming growth factor-β/Smad2 signaling pathway (9). Conversely, AECs can activate defensive mechanisms by producing antimicrobial peptides like human β-defensin 1 through the extracellular signal–regulated kinases 1/2–CCAAT/enhancer-binding protein beta pathway (10). Furthermore, Mtb infection disrupts the normal crosstalk between lung epithelial cells and antigen-presenting cells such as dendritic cells (8), emphasizing the critical role of AECs in TB pathogenesis. Nonetheless, the intricate host–pathogen interactions, as well as intercellular crosstalk within the lung epithelial microenvironment, remain largely elusive.

The *Bacillus* Calmette–Guérin (BCG) vaccine and the H37Rv strain, a well-characterized virulent laboratory strain, display distinct behaviors upon infecting macrophages from humans or mice (11, 12). H37Rv, in particular, tends to provoke more severe pathological responses. Although significant research has centered on the immune responses of macrophages to various Mtb strains (12, 13, 14), the role of AECs, especially their contribution to the virulence of potent Mtb strains within the lung epithelial niche, is still largely unexplored.

In addition, current *in vivo* and traditional *in vitro* models pose significant limitations in thoroughly investigating host–pathogen interactions and the crosstalk between AECs and peripheral blood mononuclear cells (PBMCs). *In vivo* models are often confounded by numerous systemic factors, making it challenging to isolate specific cellular interactions. Meanwhile, *in vitro* models based solely on lung epithelial cell lines lack the physiological complexity of the lung microenvironment. In this context, the organoid models, derived from 3D cultivation of stem cell differentiation and tissues, emerges as a promising tool that closely mimics the structural and functional characteristics of human organs (15). These models can significantly enhance our understanding of TB pathogenesis and facilitate the development of novel therapeutic strategies (16).

In this study, we employed an organoid coculture model to systematically investigate how Mtb remodels the alveolar epithelial microenvironment and how PBMCs respond to these alterations *via* paracrine signaling to counteract Mtb-induced pathological effects. Our RNA-Seq and functional assays identified distinct cellular responses to BCG and H37Rv infection and elucidated the critical paracrine crosstalk between organoids and PBMCs in regulating ferroptosis, a key determinant of Mtb survival. This study contributes to bridging a critical knowledge gap in our comprehensive understanding of the pathogenic and protective immune mechanisms that operate during the early stages of Mtb infection.

## Results

### Mtb preferentially infects alveolar epithelial type II cells

**Figure 1:**
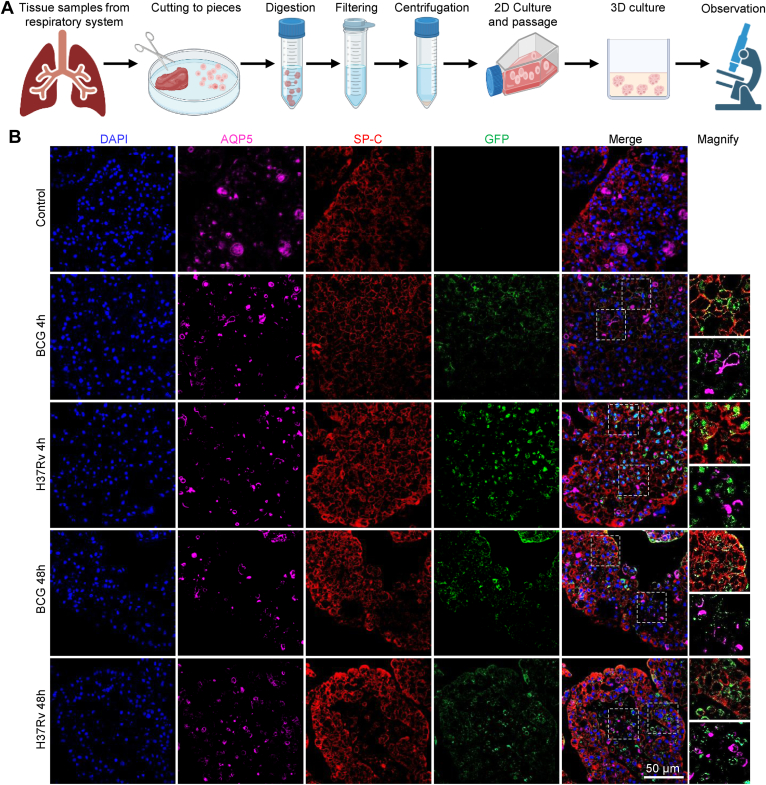
**H37Rv demonstrates high infection efficiency in AEC-II.***A,* schematic diagram of generation of human alveolar epithelial organoids. *B,* IHC staining of uninfected, BCG-infected, and H37Rv-infected organoids at 4- and 48-h postinfection. Selected regions were magnified in the *right panel*. AEC, alveolar epithelial cell; BCG, *Bacillus* Calmette–Guérin; IHC, immunohistochemistry.

To evaluate the cellular morphology and characteristics of Mtb infection, we established a human alveolar epithelial organoid coculture model following standard protocols described in the *Experimental procedures* section (Fig. 1*A*). Immunofluorescence analysis demonstrated the coexistence of AEC-I and AEC-II cells within the organoids. This was validated using lineage-specific markers: aquaporin 5 (AQP5) for AEC-I cells and surfactant protein C (SP-C) for AEC-II cells (Fig. 1*B*) (17). Subsequently, GFP staining was used to localize Mtb. Paraffin sections showed strong colocalization of BCG-GFP and H37Rv-GFP with SP-C, whereas minimal overlap was observed with AQP5, indicating that both Mtb strains show a preference for infecting AEC-II cells.

To explore the molecular mechanisms underlying host responses to different Mtb strains, we performed RNA-Seq on BCG- and H37Rv-infected organoids as well as their cocultured PBMCs. The uninfected controls were also included to discern the baseline transcriptional landscape. The t-distributed stochastic neighbor embedding (t-SNE) analysis revealed distinct transcriptional clusters corresponding to specific cell types (Fig. S1). Notably, PBMCs cocultured with Mtb-infected organoids exhibited greater transcriptional heterogeneity. These observations indicate a diverse range of molecular adaptations by immune cells within the infection-altered microenvironment.

### Mtb infection reprogrammed cell death patterns

**Figure 2:**
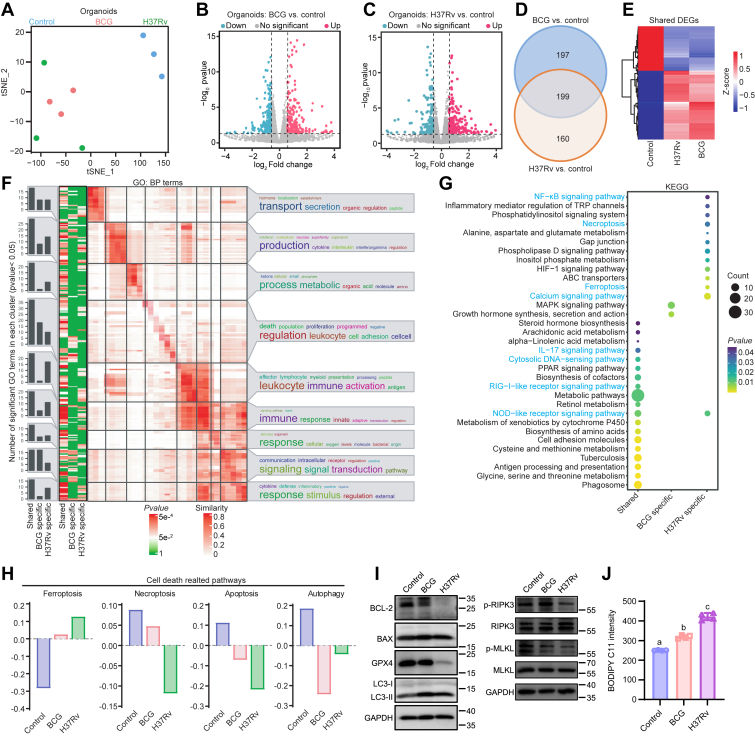
**BCG and H37Rv infections induce distinct transcription profiles in lung epithelial organoids.***A,* t-SNE visualization of global transcriptional profiles in uninfected, BCG-infected, and H37Rv-infected organoids. *B* and *C,* Volcano plots showing global transcriptional differences between BCG-infected *versus* uninfected organoids. (*B*) H37Rv-infected *versus* uninfected organoids (*C*). *D, Venn diagram* illustrating the overlap of DEGs between BCG-infected *versus* uninfected and H37Rv-infected *versus* uninfected organoids in two pairwise comparisons. *E,* heatmap displaying the expression patterns of both Mtb strains commonly regulated DEGs in uninfected, BCG-infected, and H37Rv-infected organoids. *F* and *G,* heatmap clustering of significant GO terms (*F*) and enrichment of KEGG pathways (*G*) associated with both shared and specific DEGs. *H,* GSVA score of ferroptosis, necroptosis, apoptosis, and autophagy in uninfected, BCG-infected, and H37Rv-infected organoids. *I,* Western blot of ferroptosis (GPX4), autophagy (LC3B), apoptosis (BAX, BCL2), and necroptosis (RIPK3, p-RIPK3, MLKL, and p-MLKL) in uninfected, BCG-, and H37Rv-infected organoids. *J,* assessment of lipid peroxidation levels induced by BCG and H37Rv infections *via* flow cytometric analysis using BODIPY C11 staining. BCG, *Bacillus* Calmette–Guérin; DEG, differentially expressed gene; GO, Gene Ontology; GSVA, gene set variation analysis; KEGG, Kyoto Encyclopedia of Genes and Genomes; Mtb, *Mycobacterium tuberculosis*; t-SNE, t-distributed stochastic neighbor embedding.

We then delved into the specific impacts of BCG and H37Rv infections on the transcriptional profiles of organoids. t-SNE analysis revealed a clear segregation between infected and uninfected organoids, highlighting the profound transcriptional alterations induced by both strains (Fig. 2*A*). Differential analysis showed that nearly 50% of differentially expressed genes (DEGs) were consistently impacted by both Mtb strains (Fig. 2, *B*–*E*).

To investigate the commonalities and differences in the biological functions affected by BCG and H37Rv, we performed gene enrichment analyses on strain-specific and shared DEGs. Gene Ontology enrichment categorized these genes into nine functional groups, including transport/secretion, metabolism, immune response, and signal transduction, and others (Fig. 2*F*). Kyoto Encyclopedia of Genes and Genomes (KEGG) pathway analyses revealed that shared DEGs were enriched in immune response pathways, such as DNA and RNA sensing signals (Fig. 2*G* and S2*A*). This impact was validated by expression of cytokines, with BCG infection resulting in the upregulation of tumor necrosis factor alpha (*TNFα*) (Fig. S2*B*). In contrast, H37Rv infection significantly upregulated *IL18*, whereas downregulating *IL6*, both of which are prominent proinflammatory cytokines. Moreover, H37Rv infection strongly promoted the interferon response, as evidenced by the upregulation of *IFNβ* expression. To further elucidate the mechanisms underlying H37Rv virulence, particular attention was given to pathways uniquely influenced by this strain, particularly those associated with cell death (Fig. 2, *G* and *H*). Previous studies have established a strong link between such pathways and Mtb survival and dissemination (18). Among cell death processes, ferroptosis and necroptosis were benefited for Mtb growth and dissemination. Ferroptosis, an iron-dependent form of cell death driven by lipid peroxidation, was significantly elevated in H37Rv-infected organoids (19), as evidenced by reduced GPX4 (a selenoprotein known as phospholipid hydroperoxide glutathione peroxidase, has been identified as a central suppressor of ferroptosis) and increased lipid peroxidation (Fig. 2, *I* and *J*). Similar phenotype was observed in macrophages as well (Fig. S3, *A* and *B*). Furthermore, suppressed RIPK3 and MLKL pointed to an inhibition of necroptosis. Given that NF- κB signaling is closely correlated with apoptosis, and calcium signaling is implicated in apoptosis and autophagy—processes known to restrict bacterial growth and support immune responses against Mtb—we thus investigated the cellular outcomes of these pathways (20, 21). Gene set variation analysis (GSVA) combined with Western blot experiments revealed that H37Rv infection significantly decreased the BCL2/BAX ratio, indicating an enhanced promotion of apoptosis (Fig. 2, *H* and *I*). Notably, both BCG- and H37Rv-infected organoids displayed increased LC3-II protein levels, reflecting elevated autophagic activity (Fig. 2*I*). We also investigated pyroptosis, a cell death pathway known to restrict Mtb growth (22). Our findings revealed that H37Rv infection led to a slight decrease in the N-terminal fragment of GSDMD and cleaved caspase-1 (Fig. S3*C*), suggesting a subtle inhibition of pyroptosis. Taken together, these findings suggest that H37Rv remodels host cell death pathways by promoting ferroptosis and apoptosis while concurrently suppressing necroptosis and pyroptosis.

To uncover the primary regulators driving BCG- and H37Rv-induced mRNA changes in organoids, protein–protein interaction (PPI) networks were constructed (Fig. S4). The network from BCG-specific DEGs compromised 102 nodes and 142 interactions, with the top three modules linked to cellular responses to reactive oxygen species, cAMP, and cytokines (Fig. S5, *A* and *B*). In contrast, the H37Rv network consisted of 77 nodes and 89 interactions, with its three main modules focusing on signal transduction, immune responses, and bacterial interaction mechanisms (Fig. S5, *C* and *D*). We then identified the top 10 hub genes for each network, which were further used to explore the most related genes by GeneMANIA (Fig. S5, *E* and *F*). The analysis revealed that BCG-specific hub genes, along with their associated genes, were primarily involved in innate immune response regulation, interferon signaling, and IL-27 pathways (Figs. S5*E* and 5*G*). Hub genes specific to H37Rv were linked to peptide crosslinking processes, antimicrobial humoral responses, and broader immune actions (Fig. S5*F* and 5*H*). Both GeneMANIA networks displayed a predominance of coexpression (>85%) interactions.

### Mtb infections impact signal transduction pathways

**Figure 3:**
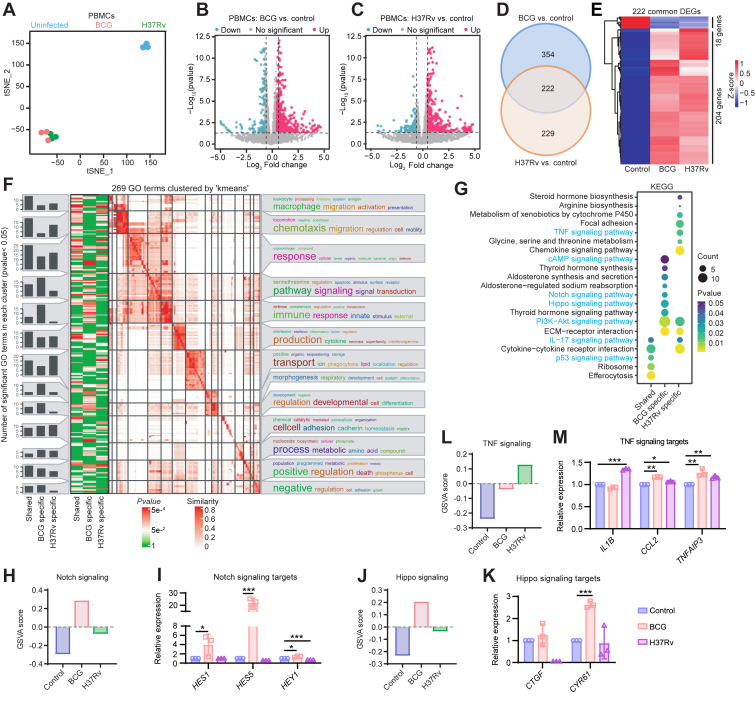
**Differential transcriptional responses of PBMCs to BCG- and H37Rv-infected organoids**. *A,* t-SNE visualization of global transcription profiles in PBMCs cocultured with BCG- and H37Rv-infected organoids, alongside uninfected controls. *B* and *C,* Volcano plots depicting global transcriptional differences of genes in PBMCs exposed to BCG-infected (*B*) and H37Rv-infected (*C*) organoids, compared with uninfected controls. *D,* Venn diagram illustrating the overlap of DEGs in PBMCs between two pairwise comparisons: BCG *versus* control and H37Rv *versus* control. *E,* heatmap showing the expression patterns of 222 shared genes in PBMCs exposed to uninfected, BCG-, and H37Rv-infected organoids. *F* and *G,* GO terms clustering (*F*) and KEGG pathways enrichment (*G*) analysis for PBMC DEGs. Both BCG- and H37Rv-specific, as well as shared impacted genes, are included in these analyses. *H*, *J,* and *L,* GSVA scores for Notch (*H*), Hippo (*J*), and TNF (*L*) signaling pathways in PBMCs. *I, K,* and *M,* qPCR analysis of target genes in the Notch (*I*), and Hippo (*K*), and TNF (*M*) signaling pathways in PBMCs. BCG, *Bacillus* Calmette–Guérin; DEG, differentially expressed gene; GO, Gene Ontology; GSVA, gene set variation analysis; KEGG, Kyoto Encyclopedia of Genes and Genomes; PBMC, peripheral blood mononuclear cell; qPCR, quantitative PCR; TNF, tumor necrosis factor; t-SNE, t-distributed stochastic neighbor embedding.

Next, we tried to investigate the transcriptional response of PBMCs to organoids infected with Mtb. t-SNE analysis revealed distinguished transcriptional patterns between Mtb-infected and -uninfected groups (Fig. 3*A*). Compared with control, differential analyses identified 576 and 451 genes were significantly affected by BCG and H37Rv, respectively (Fig. 3, *B* and *C*). Interestingly, 222 shared DEGs consistently altered in response to both infections, with 91.9% (204/222) being upregulated (Fig. 3, *D* and *E*). These DEGs primarily contributed to macrophage migration/activation, cellular responses, and cytokine production. Genes uniquely influenced by BCG showed a pronounced involvement in signal transduction, immune response, and developmental regulation, whereas H37Rv uniquely affected processes related to chemotaxis and transport (Fig. 3*F*). KEGG enrichment further illuminated the distinct functional pathways regulated by BCG and H37Rv infections, highlighting a significant focus on signal transduction pathways (Figs. 3*G*, S6, *A*–*D*). GSVA revealed that H37Rv infection intensified TNF signaling activity, whereas BCG enhanced signaling within the Notch and Hippo pathways (Fig. 3, *H*, *J* and *L*). Supporting these findings, RT–quantitative PCR (qPCR) demonstrated increased expression of Notch signaling targets (*HES1*, *HES5*, and *HEY1*) and the Hippo signaling effector *CYR61* following BCG infection, indicating the activation of these developmental signaling pathways (Fig. 3, *I* and *K*). H37Rv infection predominantly upregulated proinflammatory cytokines, such as *IL1B*, *CCL2*, and *TNFAIP3*, reflecting activation of TNF signaling (Fig. 3*M*). These results indicate that PBMCs adapt to BCG and H37Rv infections through distinct immune modulation mechanisms. BCG primarily enhances developmental signaling pathways, whereas H37Rv triggers a proinflammatory response. In addition, the activation of these pathways in PBMCs was corroborated through analysis of TB patient cohorts (Fig. S6*E*).

Further PPI network analysis displayed unique topologies. The network derived from BCG-specific regulated DEGs in PBMCs consisted of 225 nodes and 692 interactions (Fig. S7). The top three modules identified in this network were involved in interferon signaling, oxidative phosphorylation, and signal transduction (Fig. S8*A*). In contrast, H37Rv-induced network comprised 134 nodes and 303 interactions, forming eight modules, with the top three prominent modules related to chemokine response, cell periphery, and cell development (Fig. S8*B*). Hub gene analysis revealed the interferon-stimulated genes, and chemokine genes played a dominant role in BCG and H37Rv PPI network, respectively (Fig. S8, *C*–*F*). These findings emphasize the distinct regulatory responses of PBMCs to BCG and H37Rv infections, interferon-stimulated genes predominantly regulated in response to BCG and chemokine-related factors in response to H37Rv.

### BCG and H37Rv alter the organoid–PBMCs interaction landscapes

**Figure 4:**
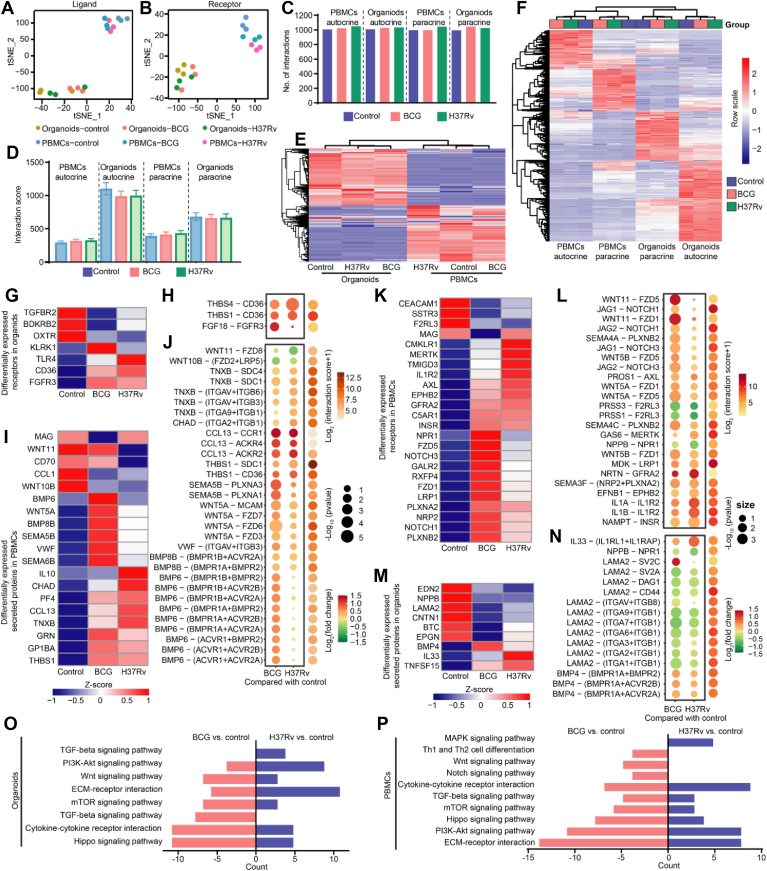
**Difference in interactions between organoids and PBMCs based on differentially expressed genes.***A* and *B,* t-SNE plots depicting the ligands (*A*) and receptors (*B*) landscapes in uninfected, BCG-infected, and H37Rv-infected organoids, as well as their cocultured PBMCs. *C, bar graph* showing the number of interactions involving autocrine and paracrine signals from PBMCs and organoids, respectively. The interaction scores >1 were considered. *D, bar plot* displaying the interaction strength of autocrine and paracrine signals from PBMCs and organoids. *E,* heatmap exhibiting the expression patterns of all ligands among uninfected, BCG-infected, and H37Rv-infected organoids, as well as their cocultured PBMCs. *F,* heatmap depicting the autocrine and paracrine patterns in uninfected, BCG-infected, and H37Rv-infected organoids, as well as their cocultured PBMCs. *G* and *K,* heatmap showing the expression patterns of DE receptors in organoids (*G*) and PBMCs (*K*) under uninfected, BCG-infected, and H37Rv-infected conditions. *H* and *L,* dot plot showing the significant ligand–receptor interactions based on DE receptors. The ligands specifically from PBMC paracrine (*H*) and organoid paracrine (*L*). *I* and *M,* heatmap showing the DE ligands in PBMCs (*I*) and organoids (*M*) under uninfected, BCG-infected, and H37Rv-infected conditions. *J* and *N, dot plot* showing the significant ligand–receptor interactions based on DE paracrine ligands. The corresponding receptors were from organoids (*J*) and PBMCs (*N*), respectively. *O* and *P, bar plots* illustrating the paracrine functions mediated by DEGs in organoids (*O*) and PBMCs (*P*), respectively. BCG, *Bacillus* Calmette–Guérin; PBMC, peripheral blood mononuclear cell; t-SNE, t-distributed stochastic neighbor embedding.

Given the distinct transcriptional profiles were observed between Mtb-infected organoids and their cocultured PBMCs, we posited that Mtb infections may influence cell–cell communication. To substantiate this hypothesis, ligand–receptor pair data were analyzed using CellChat (23). t-SNE analyses revealed notable disparities in the expression of ligands and receptors between organoids and PBMCs (Fig. 4, *A* and *B*). By quantifying the count and strength of interactions, we found that neither Mtb strain affected the total number of interactions (Fig. 4*C*), but overall, signals emitted by organoids decreased, whereas signals from PBMCs increased (Fig. 4*D*). Moreover, organoids and PBMCs displayed distinct secretion patterns, with organoids exhibiting robust autocrine signaling (Fig. 4, *E* and *F*).

We then focused on DEGs identified previously to enhance our comprehension of altered ligand–receptor interactions. Specifically, (1) receptor analysis: we retained differentially expressed (DE) receptors but excluded complex receptors where only a single subunit exhibited differential expression; (2) ligand filtering: we prioritized ligands that were not only DE in a specific cell type but also predominantly expressed in that cell type. Applying these criteria, 7 of 17 organoid DE receptors and 19 of 48 PBMC DE ligands emerged as high-confidence candidates (Fig. 4, *G* and *I*, S7, *A* and *B*). Pairwise comparisons revealed that the THBS4–CD36 interaction was consistently upregulated in both Mtb-infected organoids, whereas FGF18–FGFR3 and THBS1–CD36 interactions were uniquely upregulated in BCG- and H37Rv-infected organoids, respectively (Fig. 4*H*). In addition, BCG specifically induced the upregulation of BMP6, BMP8B, and WNT5A, whereas H37Rv specifically induced upregulation of TNXB. These interactions enhanced the paracrine signals of PBMCs (Fig. 4*J*). Pathway enrichment analyses further elucidated that BCG-induced DEG-mediated interactions were involved in WNT, mTOR, Hippo signaling, and transforming growth factor-β signaling in organoids, whereas H37Rv predominantly affected PI3K–Akt signaling (Fig. 4*O*). Focusing on the impact of DE receptors in PBMCs and ligands from organoids on PBMC functions, we identified 24 of 39 DE receptors and 9 of 13 DE ligands (Fig. 4, *K* and *M*, S7, *C* and *D*). These receptors mediated 24 significant paracrine signals from organoids, most of which were upregulated (Fig. 4*L*). In contrast, except for the upregulation of IL33- and BMP4-mediated interactions, most of the paracrine signals mediated by DE ligands from the organoids were decreased (Fig. 4*N*). These BCG-induced interaction change participated in Th1 and Th2 cell differentiation as well as WNT, Notch, and PI3K–Akt signaling in PBMCs. Conversely, H37Rv predominantly impacted mitogen-activated protein kinase signaling in PBMCs (Fig. 4*P*).

**Figure 5:**
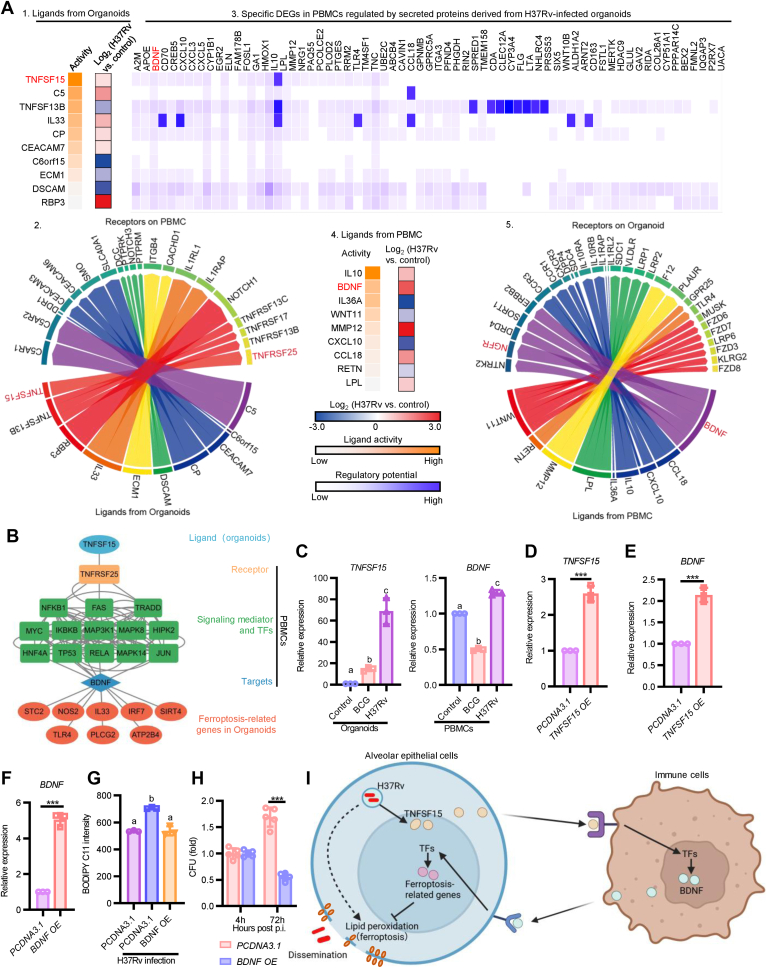
**NicheNet analysis reveals TNFSF15 and BDNF as key paracrine signals mediating ferroptosis through intercellular crosstalk during H37Rv infection.***A,* NicheNet analysis predicting upstream ligand–receptor interactions that induce the observed DEGs in H37Rv-infected PBMCs and organoids. In 1, are the potential upstream ligands from organoids, whose expression is regulated by H37Rv infection; in 2, are the potential receptors present on the surface of PBMCs, which are predicted to interact with the ligands secreted by organoids; in 3, are the potential target genes within PBMCs that are regulated by the ligands originating from organoids; in 4, are the potential upstream ligands from PBMC response to signals emanating from organoids, along with their potential activity in regulating target gene expression within organoids; in 5, are the potential receptors on the surface of organoids, which are predicted to interact with ligands from PBMCs. *B,* schematic representation of intercellular crosstalk between PBMCs and organoids mediated by ligand–receptor pairs. *C,* quantitative real-time PCR (qPCR) validation of *TNFSF15* expression in organoids and *BDNF* expression in PBMCs. *D,* the overexpression efficiency of *TNFSF15* in U937 cells. *E,* expression of *BDNF* in *TNFSF15*-overexpressing cells. *F,* the overexpression efficiency of *BDNF* in A549 cells. *G,* levels of lipid peroxidation in *BDNF*-overexpressing cells during H37Rv infection. *H,* colony-forming units (CFUs) of H37Rv in *BDNF*-overexpressing cells. *I,* schematic diagram illustrating the TNFSF15- and BDNF-mediated crosstalk between organoids and PBMCs, and its role in regulating ferroptosis during H37Rv infection. BDNF, brain-derived neurotrophic factor; DEG, differentially expressed gene; PBMC, peripheral blood mononuclear cell.

Finally, we investigated how H37Rv-infected organoids influence PBMC transcription and how PBMCs, in turn, respond to this altered microenvironment. For this purpose, we employed NicheNet, a computational framework widely used to reconstruct signaling cascades from ligand–receptor interactions to downstream transcriptional responses (24, 25, 26). Using this method, we demonstrated that H37Rv infection resulted in the upregulation of TNFSF15 (a member of the TNF superfamily) in organoids (Fig. 5, *A* and *B*). Subsequently, this organoid-derived TNFSF15 was found to interact with the TNFRSF25 receptor on PBMCs, leading to the induction of BDNF expression. Intriguingly, BDNF secreted by PBMCs then establishes a feedback loop by binding to cognate receptors on the organoids, thereby modulating genes critical for ferroptosis regulation. To functionally validate this network, we first examined the expression levels of *TNFSF15* and *BDNF*. RT–PCR showed that *TNFSF15* was indeed upregulated in H37Rv-infected organoids, and a corresponding increase in *BDNF* expression was also observed in PBMCs (Fig. 5*C*). Interestingly, TNFSF15 overexpression enhanced BDNF expression (Fig. 5, *D* and *E*). To further elucidate the effects of BDNF on ferroptosis and Mtb growth, we overexpressed BDNF in A549, a cell line commonly used model for AEC-II (27, 28). Following H37Rv infection, we found BDNF overexpression not only inhibited ferroptosis but also restricted Mtb growth (Fig. 5, *F*–*H*). These results suggest that organoid–PBMC crosstalk plays an essential role in aiding host defense against virulent Mtb strain.

## Discussion

AECs provide a niche for Mtb replication and dissemination during early infection and directly interact with alveolar macrophages to regulate cytokine expression in response to pathogens (4, 5). This reinforces the idea that lung epithelial cells are important for dissemination and/or control of Mtb. Despite its significance, the understanding of pathogen–AEC interaction, especially cell crosstalk within the lung microenvironment, remained largely limited. In this study, we leveraged an organoid coculture model, which closely mimics the *in vivo* environment. Through RNA-Seq and functional experiments, we explored the landscape of host–pathogen interactions specific to the lung epithelial microenvironment.

Cell death patterns are directly linked to Mtb survival and dissemination. While prior studies have shown that virulent H37Rv can induce ferroptosis in macrophages, promoting bacterial spread, and that ferroptosis inhibition reduces bacterial loads (*e.g.*, ferrostatin-1 treatment) (29, 30), our study extends this to AECs. We demonstrate that both BCG and H37Rv infection induce ferroptosis in AECs, specifically AEC-II cells, by downregulating GPX4 and promoting lipid peroxidation. Notably, H37Rv exhibited a more potent induction of ferroptosis compared with BCG, a finding consistent with previous observations across different models and cell types (31, 32). Similar to ferroptosis, necroptosis also benefits to Mtb growth. This form of cell death depends on RIPK1 and RIPK3 signaling pathways and is regulated by TNF (33, 34). However, our coculture studies reveal that H37Rv infection reduces p-RIPK3 and p-MLKL, suggesting that this virulent Mtb strain does not rely on necroptosis to promote its growth. Our findings also show that while H37Rv and BCG both upregulate autophagic flux and H37Rv promotes apoptosis, these processes may contribute to limiting Mtb survival rather than promoting it in this context. Although our RNA-Seq data did not reveal obvious transcriptional alterations in pyroptosis-related genes induced by either H37Rv or BCG, we observed H37Rv infection results in a moderate inhibition of pyroptosis. This suggests that, independent of transcriptional regulation, H37Rv could potentially enhance its pathogenesis through pyroptosis. Totally, these findings suggest that virulent Mtb may enhance its pathogenicity primarily by inducing ferroptosis in lung epithelial microenvironment.

The observed differences in cell death patterns between BCG- and H37Rv-infected organoids likely stem from the significant genetic disparities between these two Mtb strains. BCG, an attenuated vaccine strain, carries genetic mutations reducing its virulence and immunogenicity, affecting its replication efficiency and survival capacity (35). By contrast, H37Rv contains 14 unique regions of difference (RD1–14), encoding virulence effectors and secretion systems. Notably, the ESX-1 secretion system enables H37Rv to actively modulate host cell death pathways and immune responses (36). This is exemplified by the secretion of proteins, such as PtpA (32), PPE10 (37), PPE51 (38), and PtpB (39), Zmp1 (40), Rv3364c (41), and PtpB (22), which have been shown to intricately manipulate cell death patterns to facilitate persistence. These findings collectively illustrate the sophisticated strategies employed by H37Rv to reprogram cellular death pathways and immune responses, thereby promoting its survival.

Despite these strategies employed by virulent Mtb to manipulate host immune evasion and cell death pathways, our study provides novel insights into host defense mechanisms within the lung epithelial microenvironment (Fig. 5*I*). Distinct from traditional ligand–receptor interactions of TB microenvironment, our NicheNet interaction network captured downstream consequences of cell crosstalk (42, 43). A key finding was the upregulation of TNFSF15 in H37Rv-infected organoids. Although previous research has not yet established a direct association between *TNFSF15* and the pathogenicity of TB, other members of TNF superfamily members (*e.g.*, TNFSF13B) are linked to TB severity (42). Notably, *TNFSF15* leads to increased *BDNF* expression in PBMCs. BDNF has well-documented roles beyond the nervous system, including modulation of immune cell function, cell death mechanisms, and protective effects against ferroptosis in neurological disease models (44, 45). Our findings expand these insights to infectious diseases, demonstrating that *BDNF* can regulate ferroptosis-related gene expression, thereby enhancing host defense mechanisms against Mtb.

Furthermore, PBMCs demonstrated distinct response patterns to infection with different Mtb strains. The dynamics of PBMC subpopulations, including CD4+ T cells (particularly Th1 and Th17), CD8+ T cells, B cells, natural killer cells, and monocytes, are crucial determinants response to Mtb infection (46). Although we did not measure the proportions of these subpopulations, pathway analyses revealed significant changes in signaling cascades, such as TNF, Notch, PI3K–Akt, cAMP, IL-17, and Hippo pathways, which influence PBMC functions. PI3K–Akt signaling promotes immune cell survival, proliferation, and cytokine production, thereby regulating T-cell and B-cell differentiation (47). Hippo signaling, crucial for T-cell development and function, is activated during Mtb invasion and modulates host immune responses by regulating chemokine expression (48). TNF signaling enhances T-cell responses and regulates chemokine expression, crucial for immune cell recruitment and maintenance at the infection sites (49). cAMP signaling influences T-cell activation/differentiation and the Th1–Th2 balance (50). Notch signaling is correlated with proinflammatory responses (51). IL-17, produced by Th17 cells, NKT cells, and CD8+ T cells, stimulates antimicrobial peptide and chemokine production, recruiting neutrophils and macrophages to the infection sites (52). While both Mtb strains affect these pathways, the extent of their activation highlights the differences between the two strains. BCG prominently enhances Notch and Hippo signaling, potentially contributing to activating specific host signaling pathways that induce immune protection rather than causing severe pathological reactions. In contrast, H37Rv may underlie a stronger proinflammatory response and associated immunopathology. Despite their significance, excessive immune responses in PBMCs can lead to cytokine storms and immune paralysis, a phenomenon that has been observed in TB patients, particularly in those with severe disease who exhibit inflammatory cytokine storms. Thus, balancing the cellular interactions between immune cells and Mtb-infected cells may be a therapeutic target (53).

While the human alveolar epithelial organoid coculture model offers valuable insights into immune–epithelial interactions during early Mtb infection, it fails to fully mimic the *in vivo* microenvironment. The inability of PBMCs to traverse the transwell and access the organoid lumen, along with the simplified three-dimensional tissue architecture, which restricts the study of crucial aspects of the immune response, including immune cell migration, epithelial barrier function, and complex cellular interactions. We also acknowledge the inherent limitations of our single-time point RNA-Seq analysis. This approach may not fully capture the dynamic temporal changes and complex regulatory mechanisms occurring during the infection, particularly concerning critical post-transcriptional modifications. Future research should incorporate time-course RNA-Seq experiments to elucidate dynamic changes in gene expression and employ complementary techniques, such as liquid chromatography–mass spectrometry, to investigate post-transcriptional regulation. Furthermore, characterizing specific Mtb effectors that interact with host regulators and assessing their impact on cell death dynamics, which is paramount for a comprehensive understanding of Mtb pathogenesis. Despite these limitations, the organoid coculture model, associated RNA-Seq dataset, observed cell death patterns, and the established PBMC–AEC-II crosstalk models presented in this study provide invaluable resources for researchers aiming to deeply understand Mtb's pathogenic mechanisms and to develop novel therapeutic strategies during early infection.

### Conclusion

In summary, through depicting the organoids and PBMC transcriptional profiles, as well as their interaction landscape, we disclosed intricate dynamics between the host and the pathogen during Mtb infection. The disparities discerned between BCG and H37Rv strains provide invaluable insights not only into the distinct genetic signatures elicited by different Mtb strains but also into the underlying mechanisms that may explain the variability observed in disease progression and immune responses among TB patients.

## Experimental procedures

### Human lung organoid culture

Human lung tissues were obtained from surplus samples of surgically removed lung tissue from patients with interstitial lung diseases requiring surgery. The patient had no significant respiratory symptoms (*e.g.*, cough, dyspnea) for at least 1 year prior to surgery and no long-term use of immunosuppressive agents, antifibrotic drugs, or other therapies related to interstitial lung disease. Meanwhile, the patient had no history of smoking, occupational exposure, radiotherapy, or chemotherapy. Informed consent was obtained from donors or their authorized representatives for all specimens. The tissues were washed with PBS, cut into pieces, and digested with an enzyme mixture (1.5 mg/ml collagenase type II; 0.5 mg/ml dispase; and 10 U/ml DNase I) at 37 °C for 15 min. The cells were then filtered through a 100 μm strainer and rinsed with Dulbecco's modified Eagle's medium (DMEM)/F-12 medium. After centrifugation, the supernatant was discarded, and the cell pellet was resuspended in red blood cell lysis buffer (bioGenous; E238010) for 5 min. Following a wash with DMEM/F-12 medium, the total cells were centrifuged at 350*g* for 5 min at 4 °C. Epithelial cells were seeded into T25 cell culture flasks containing PneumaCult-Ex Plus Medium (StemCell Technologies; catalog no.: 05040). When the cells reached 50 to 70% confluency, they were harvested and seeded in 24-well AggreWell 400 plates (StemCell Technologies; catalog no.: 34421). The AggreWell plates were centrifuged at 200*g* for 3 min to sediment and aggregate the cells to the bottom of each microwell. Lung epithelial cells were differentiated for a total of 15 days.

### Cell culture

A549 and RAW264.7 cell lines were maintained in DMEM (catalog no.: D629; Gibco) supplemented with 10% fetal bovine serum. U937 cells were cultured in RPMI1640 (catalog no.: R8758; Gibco) containing 10% fetal bovine serum. To induce differentiation into adherent macrophage-like cells, U937 cells were stimulated with 10 ng/ml phorbol 12-myristate 13-acetate overnight. Following differentiation, cells were washed once with PBS and subsequently cultured in fresh RPMI1640 medium.

### Bacterial culture and infection

Mtb was cultured in Middlebrook 7H9 broth (BD Biosciences; catalog no.: 271310) supplemented with 0.5% glycerol (Sangon Biotech; catalog no.: A100854), 0.05% Tween-80 (Sangon Biotech; catalog no.: A458113), and 10% oleic acid–dextrose–catalase (BD Biosciences; catalog no.: 212240) at 37 °C. After 2 to 3 weeks, bacteria in midlog phase (absorbance at 600 nm = 0.6–0.8) were centrifuged at 3750 RPM for 10 min, then resuspended in PBS (Thermo Fisher Scientific; catalog no.: 10010023) containing 0.05% Tween-20 (Sangon Biotech; catalog no.: A600560) and 25% glycerol, and stored at −80 °C. To determine the exact number of colony-forming units of Mtb, a vial of the frozen stock was thawed and diluted to calculate colony-forming unit per milliliter. Prior to infection, the frozen bacteria were thawed and centrifuged at 12,000 RPM for 2 min, then resuspended in 1640 culture medium (Gibco; catalog no.: 11875119). The organoids were infected with Mtb at a multiplicity of infection of 10. Following 4 h of incubation, the organoids were thoroughly washed three times with PBS. Subsequently, fresh 1640 medium was added for further culture.

### PBMC collection and cocultured with organoids

On the day of coculture experiment, a healthy volunteer from the Shenzhen Third People's Hospital, adhering to a healthy lifestyle and free from recent illness, infectious diseases, and chronic conditions, was recruited with informed consent. Using a sterile bag, 100 ml of whole blood was collected. PBMCs were isolated by centrifuging the blood at 2800 RPM for 10 min to separate components. The buffy coat, enriched with white blood cells, was harvested, diluted with PBS, and layered over Ficoll–Paque Plus. After centrifugation at 3000 RPM for 20 min, mononuclear cells were isolated based on density. The cells undergone two PBS washes, erythrocyte lysis, and another wash to remove contaminants and lysis buffer. Finally, PBMCs were resuspended in RPMI1640 medium and counted. For coculture, PBMCs were added to the bottom wells of a 24-well plate at a 10:1 ratio to infected organoids. The coculture was incubated at 37 °C under 5% CO_2_ until the desired harvest endpoint.

### Immunohistochemistry staining

Immunohistochemistry staining was applied to the lung organoids to identify the indicated cell types and Mtb-infected cells. Paraffin sections were first dewaxed and rehydrated before antigen retrieval. Antigen retrieval was performed using 10 mM sodium citrate (pH 6.0) in a water bath at 95 °C for 15 min. Sections were washed with 0.1% Triton X-100 in PBS (PBST) and incubated with 1% bovine serum albumin for 10 min at room temperature, followed by primary antibodies for 30 min at room temperature or overnight at 4 °C. The specific primary antibodies were used to recognize alveolar type I cells (AEC-I, AQP5), alveolar type II cells (AEC-II, SP-C), and M.tb strains (GFP). Sections were washed three times with PBST for 5 min each, incubated with secondary antibodies for 10 min at room temperature, washed with PBST three times more, and then incubated with fluorescent signal amplification reaction buffer. Secondary antibodies and fluorescent signal amplification reaction buffer were used according to the Novo-Light 4-color multiplex fluorescent Immunohistochemistry kit instructions (WiSee Biotechnology; catalog no.: H-D110041). Confocal images were acquired using a Carl Zeiss LSM 900 confocal microscope.

### Protein extraction and Western blot

Organoids were collected into 1.5 ml tubes and washed three times with precooled PBS. Then, RIPA Complete Lysis Buffer (catalog no.: P0038; Beyotime) supplemented with protease and phosphatase inhibitor (catalog no.: P1045; Beyotime) was added. After lysed on ice for 10 min, the total protein was extracted and the concentration was detected using BCA Protein Assay Kit (catalog no.: P0012; Beyotime). The extracted proteins were separated by SDS-PAGE and transferred to polyvinylidene fluoride membranes. Membranes were then blocked with TBST containing 5% dry milk for 1 h at room temperature and incubated overnight at 4 °C with diluted primary antibodies against GPX4 (catalog no.: Ab125066; abcam, 1: 2000 dilution), RIPK3 (catalog no.: 13526S; CST, 1:2000 dilution), p-RIPK3 (catalog no.: 93654S; CST, 1:2000 dilution), MLKL (catalog no.: 14993S; CST, 1:2000 dilution), p-MLKL (catalog no.: 91689S; CST, 1:2000 dilution), BAX (catalog no.: 50599; Proteintech, 1:3000 dilution), BCL2 (catalog no.: 26593; Proteintech, 1:3000 dilution), LC3B (catalog no.: L8918; Sigma, 1:1000 dilution), N-GSDMD (catalog no.: 10137S; CST, 1:2000 dilution), Cleaved Caspase-1 (catalog no.: 89332S; CST, 1:1000 dilution), and GAPDH (catalog no.: 4970L; CST, 1:5000 dilution). On the second day, the membranes were washed three times with TBST and incubated with the horseradish peroxidase–conjugated secondary antibodies for 1 h. Finally, the immunostained protein bands were captured with ChemiDoc MP imaging system (Bio-Rad). The protein level was quantified by ImageJ (NIH).

### RNA extraction and real-time qPCR analysis

Total RNA was extracted from samples using TRIzol reagent (Invitrogen; catalog no.: 15596018CN). The extracted RNA was reverse-transcribed into complementary DNA (cDNA) using the HiScript II Q Select RT SuperMix for qPCR kit (catalog no.: R233-01; Vazyme). qPCR was then performed using the synthesized cDNA as a template and the ChamQ Universal SYBR qPCR Master Mix (catalog no.: Q711-02; Vazyme). GAPDH was employed as an internal control for normalization. Relative gene expression levels were determined using the 2^−ΔΔCt^ method. Primer sequences for all genes are listed in Table S1.

### Flow cytometry analysis

Lipid peroxidation was tested using the BODIPY 581/591 C11 probe (catalog no.: D3861; Thermo Fisher Scientific) according to the manufacturer's instructions. Briefly, organoids and cells were incubated with C11-BODIPY (10 μM) for 30 min at 37 °C. After incubation, cells and organoids were washed three times with precooled PBS and digested with 0.25% trypsin (catalog no.: 25200056; Thermo Fisher Scientific). The resulting cell suspensions were resuspended in fresh PBS containing 0.1% bovine serum albumin. Fluorescence was captured on a BD FACSCanto (BD Biosciences) and analyzed with FlowJo software (Becton, Dickinson & Compan).

### NicheNet analysis

The NicheNet analysis was used to profile the regulatory mechanisms of cell-to-cell crosstalk on cellular gene expression based on nichenetr package. Briefly, ligand activity prediction was performed by integrating DE ligands in sender cells with their cognate receptors' expression profiles in receiver cells, utilizing curated ligand–receptor interaction databases. Following this, potential target genes were prioritized based on the DEGs within the receiver cells, relative to a comprehensive background gene set. The resulting interaction networks and data were subsequently visualized using the circlize package and Cytoscape software (Cytoscape Consoritum).

### Plasmid construction

Overexpression plasmids for *TNFSF15* and *BDNF* were constructed by cloning the target sequences from a human cDNA library using 2× Phanta Flash Master Mix (P520; Vazyme). The amplified fragments were subsequently cloned into the pcDNA3.1(+) vector through homologous recombination. Proper integration of recombinant sequences was verified through sequencing analysis. The validated plasmids were then transfected into cells by using Lipofectamine 3000 (catalog no.: L3000015; Invitrogen) according to the manufacturer's instructions. The recombinant sequences are included in Table S1.

### Measurement of malondialdehyde level

The levels of malondialdehyde (MDA) were determined using the Lipid Peroxidation (MDA) Assay Kit (catalog no.: MAK568; Sigma) following the manufacturer's protocol. In brief, cell lysate supernatants and a series of MDA standard samples were combined with the MDA working solution. The resulting mixtures were then boiled at 100 °C for 15 min. After cooling to room temperature, the samples were aliquoted into a 96-well plate, and their absorbance was acquired at 532 nm using a Varioskan LUX spectrophotometer (Thermo Fisher Scientific). MDA concentration was subsequently expressed as μmol/mg protein.

### RNA extraction, cDNA library construction, and RNA-Seq

After 24 h of coculture, organoids were thoroughly washed three times with PBS, and TRIzol (Invitrogen; catalog no.: 15596018CN) was added to extract RNA. Similarly, PBMCs were carefully scraped off, collected, and centrifuged at 5000 RPM for 5 min to collect the cells. After washed three times, the cells were resuspended in TRIzol for RNA extraction. The total RNA was extracted according to the manufacturer's instructions.

RNA integrity was quantified on Agilent 2100, and samples with RNA Integrity Number greater than 6 were suitable. RNA purity was assessed with a Nanodrop ND-2000 spectrophotometer, with acceptable samples exhibiting A260:A280 >1.8 and A260:A230 >2.0. To prepare the samples for sequencing, ribosomal RNAs were removed using Ribo-Zero rRNA Removal Kits (Illumina) according to the manufacturer's protocol. Subsequently, cDNA libraries were constructed and sequenced on Illumina HiSeq 4000 with pair-end 150 bp strategy.

### Transcriptome assembly and differential gene analysis

After removing the adapters and low-quality reads, the clean reads were aligned to the human genome (hg38) using HISAT2 software (https://ccb.jhu.edu/software/hisat2). The transcriptome was then assembled with StringTie and potentially annotated using Cuffcompare from the Cufflinks package. The expression levels for all transcripts were quantified using fragments per kilobase of exon model per million mapped reads. Differential analyses between two groups were performed using the DESeq2 package in R, with significant changes defined by |fold change| >1.5 and *p* < 0.05.

### t-SNE analysis

t-SNE was performed with Rtsne package in R.

### Enrichment analysis

Gene Ontology enrichment analysis was conducted using the Database for Annotation, Visualization, and Integrated Discovery (http://david.abcc.ncifcrf.gov). Terms with a *p* value <0.05 were further grouped into clusters and visualized using the “simplifyEnrichment” package in R. For KEGG enrichment analysis, the genes of interested were input into WebGestalt (http://webgestalt.org). Pathways with a *p* value <0.05 were identified and visualized using the “ggplot2” package in R.

### PPI network construction, MCODE analysis, hub gene identification, and GeneMANIA analysis

The PPI network was constructed using the STRING database. Subnetworks were identified using the MCODE module in Cytoscape. The top 10 hub genes in the network were identified using the Maximal Clique Centrality algorithm in the CytoHubba app. GeneMANIA prioritized genes correlated with hub genes, leveraging its capabilities for gene function prediction, analysis, and sequencing.

### Pathway activity analyzation

The gene sets of interested pathways were downloaded from the KEGG database. GSVA was performed using the R package GSVA to estimate pathway activities. Additionally, differential analysis of KEGG pathways was conducted using the R package “limma”, with significance based on *p*-value < 0.05. Finally, GraphPad software was employed to visualize the results of these analyses.

### Pathway validation in published literature

To validate our results, transcriptome datasets of PBMCs (GSE83456, GSE62525, GSE147964, and GSE28623) from the National Center for Biotechnology Information were downloaded, including healthy controls and TB patients. After normalization, differential expression analysis was performed using DESeq2 or limma, comparing TB patients to healthy controls. Pathway activities were estimated by GSVA.

### Cell–cell communication analysis

To accurately quantify the impact of BCG and H37Rv infections on cell communication occurring between organoids and PBMCs, as well as within these cells themselves, we retrieved 1939 interaction pairs from CellChat. To quantify the number of paracrine and autocrine signals emitted by PBMCs and organoids, we first filtered out those interaction pairs where any component—a ligand, a receptor, or a subunit within a receptor complex—had a fragment per kilobase of exon model per million mapped read value of 0. Then, we calculated the interaction scores for the remaining pairs, considering only those with an interaction score >1.

Based on previous reports (54, 55), the interaction scores for a given ligand–receptor pair were determined as the square root of the product of the averages of the ligand and receptor expression levels, which can be formulated as:

$${\text{LR}}_{\text{score}}=\sqrt[{2}]{\frac{1}{3}\times \sum_{i=1}^{3}\text{Li}\times \frac{1}{3}\times \sum_{j=1}^{3}\text{Rj}}$$

Li = expression of ligand.

Rj = expression of receptor.

If a receptor consisted of n subunits, the receptor expression was defined as the geometric mean of the expression value of all subunits, the formula as follows:

$$R=\sqrt[{n}]{\prod_{j=1}^{n}\text{Rh}}$$

Rh is the expression of subunit j in the receptor complex.

To identify the primary paracrine and autocrine interactions, we performed hierarchical clustering (using the hclust function in R) and employed the cutree function to divide the interactions into four clusters (*k* = 4). These clusters were assigned to four groups: organoid paracrine, PBMC paracrine, organoid autocrine, and PBMC autocrine, based on the interaction scores. We then extracted the interaction pairs from each cluster and visualized them using the pheatmap package in R.

To identify significantly changed interactions, we performed *t* test on the interaction scores between each pair of the three repeated samples. We defined interactions with a *p* < 0.05 as significant.

### Statistical analysis

For two-group comparison, two-tailed test was used for normally distributed data. In cases of unequal variances or nonparametric data, the Welch's correction and Mann–Whitney tests were used. In scatter plots, one point represents one biological replicate.

## Data and code availability

Raw transcriptomic data were uploaded in China National Center for Bioinformation-National Genomics Data Center (accession number: HRA008967). The code reported in this article will be shared by the lead contact upon request.

## Supporting information

This article contains supporting information (Figs. S1–S9 and Table S1).

## Ethics statement

The human sample reported in this study was conducted in accordance with the ethical standards of the institutional research committee and the 1964 Declaration of Helsinki, its subsequent amendments, or comparable ethical standards. This study was reviewed and approved by the Medical Ethical Committee of Shenzhen Third People's Hospital (approved number: 2021-016-02).

## Conflict of interest

The authors declare that they have no conflicts of interest with the contents of this article.
