# Supplementary material for: Modeling Mycobacterium tuberculosis pathogenesis in lung epithelial organoids reveals strain-specific host responses and intercellular crosstalk
Source: J Biol Chem. 2025 Jul 28;301(9):110534. doi: 10.1016/j.jbc.2025.110534 (PMC12423419; doi:10.1016/j.jbc.2025.110534)
Supplement: Supporting information [file mmc1.docx]

Supplementary Materials for

**Modeling Mycobacterium tuberculosis Pathogenesis in Lung Epithelial Organoids Reveals Strain-Specific Host Responses and Intercellular Crosstalk**

*Ruiqi Zhang, Fusheng Yao, Yanhong Huang, Wenqi Liu, Shumin Liao, Junyan He, Siqi Li, Zhaoqin Wang, Liang Li, Guoliang Zhang*

Correspondence to: lil@sustech.edu.cn; zhanggl2020@mail.sustech.edu.cn

**This file includes:**

Figures. S1 to S9 and Table S1


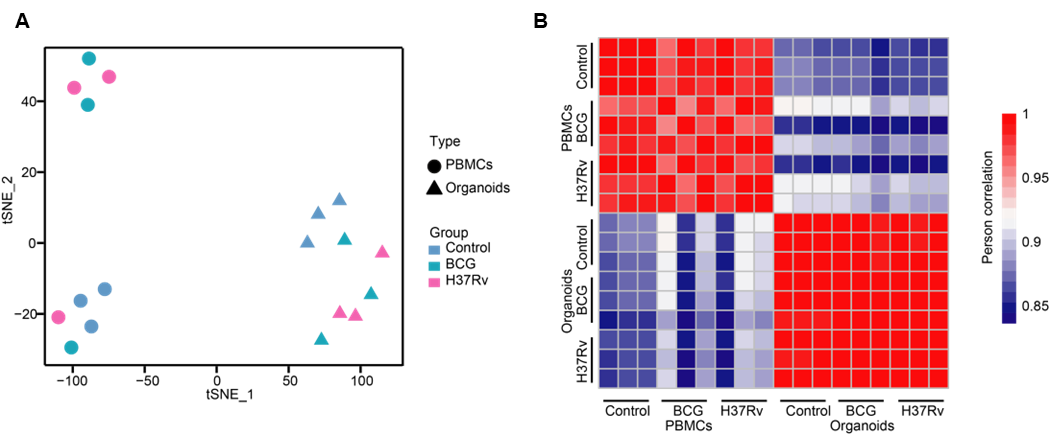


**Supplementary figure 1.** Organoids and PBMCs displaying distinct transcription profiles. (A) t-SNE visualization of global gene expression profiles in uninfected, BCG-infected organoids and H37Rv-infected organoids, their co-cultured PBMCs also included. (B) Heatmap illustrating the Pearson correlations between samples.

**Supplementary figure 2.** The impact of Mtb infections on immune response in organoids.

(A) GSVA scores of immune-related KEGG pathways in uninfected, BCG- and H37Rv-infected organoids. (B) Quantitative real-time PCR of immune-related genes in uninfected, BCG- and H37Rv-infected organoids.

**Supplementary figure 3.** The impact of Mtb infections on ferroptosis and pyroptosis. (A and B) The impact of Mtb infections on ferroptosis in RAW264.7 (A) and U937 (B) cells. (C) The impact of Mtb infections on pyroptosis in organoids.


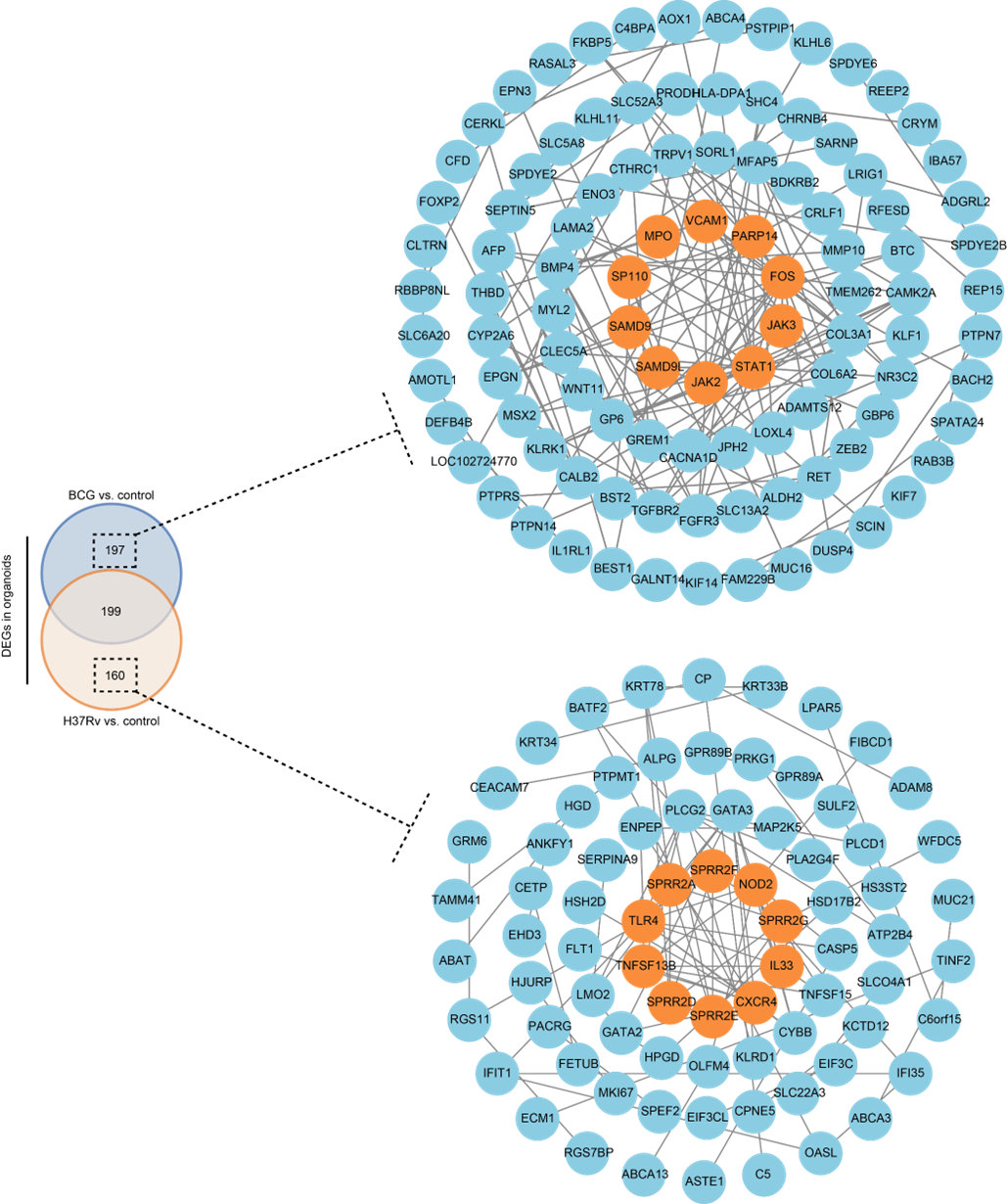


**Supplementary figure 4.** PPI networks of BCG and H37Rv specifically regulated DEGs in organoids.

**Supplementary figure 5**. Comprehensive analysis of bub genes in organoids infected with BCG and H37Rv.

(A and C) The top three MCODE subnetworks derived from BCG (A) and H37Rv (C) specifically PPI network, respectively. (B and D) The GO terms enriched within the top three MCODE subnetworks for BCG (B) and H37Rv (D) infections. (E and F) PPI networks showing BCG (E) and H37Rv (F) regulated hub genes, their most related genes, as well as potential functions. (G and H) Heatmap showing the expression patterns of BCG (G) and H37Rv (H) hub genes across uninfected, BCG-infected, and H37Rv-infected organoids.

**Supplementary figure 6.** The impact of Mtb infections on signaling pathway in PBMCs.

(A-D) GSVA scores of significantly enriched KEGG pathways in PBMCs. (B) Comparisons of pathway activities in PBMCs between TB patients and healthy controls.


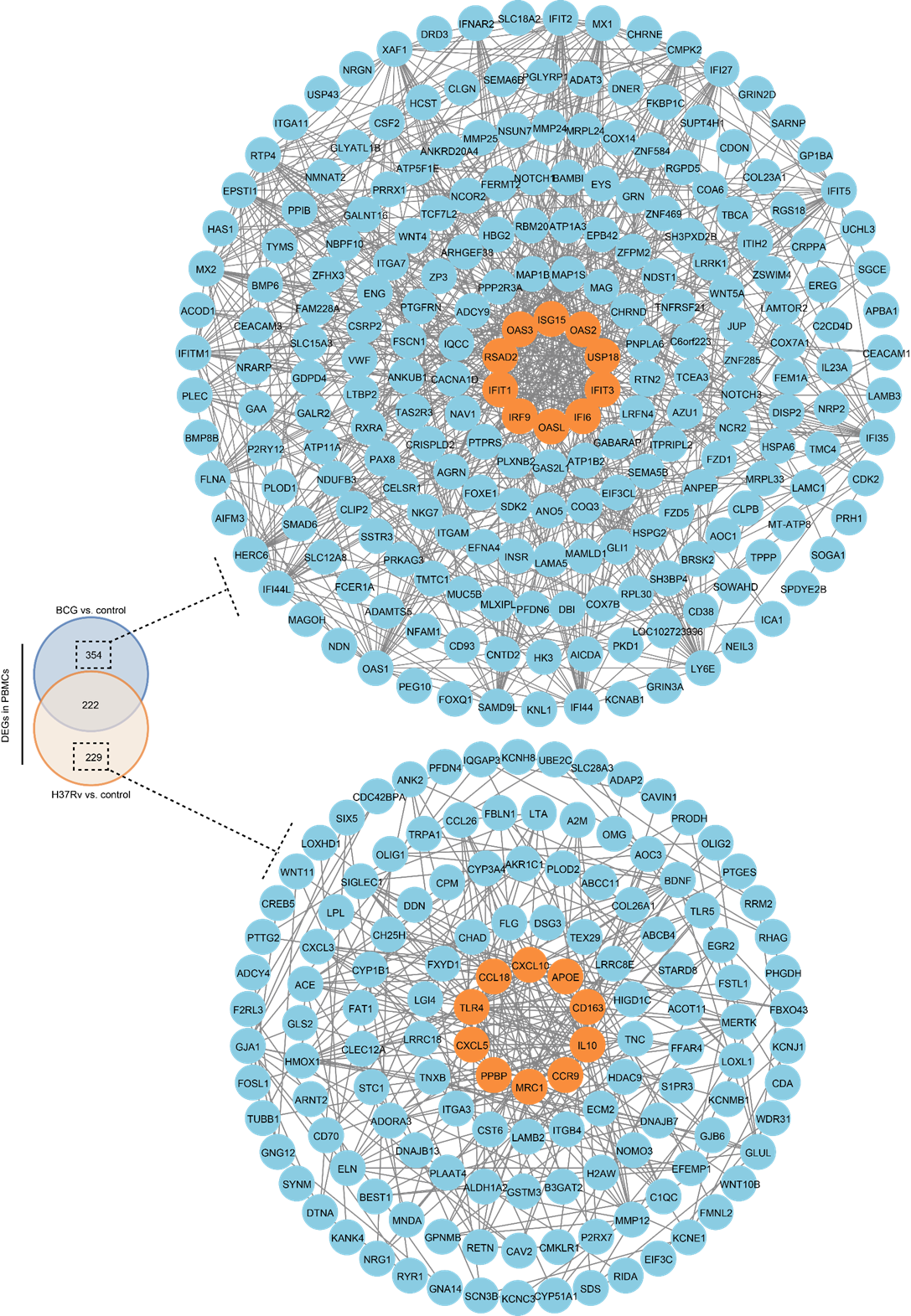


**Supplementary figure 7.** PPI networks of BCG and H37Rv specifically regulated DEGs in PBMCs.

**Supplementary figure 8**. Identification and characterization of hub genes in PBMCs exposed to BCG- and H37Rv-infected organoids.

(A and B) MCODE subnetworks derived from BCG (A) and H37Rv (B) specifically regulated DEGs in PBMCs. With threshold of score > 4. (C and D) PPI networks generated by GENEMANIA, centered on BCG (C) and H37Rv (D) regulated hub genes in PBMCs. (E and F) Heatmap showing the expression patterns of BCG (E) and H37Rv (F) hub genes in PBMCs exposed to uninfected, BCG-infected, and H37Rv-infected organoids.

**
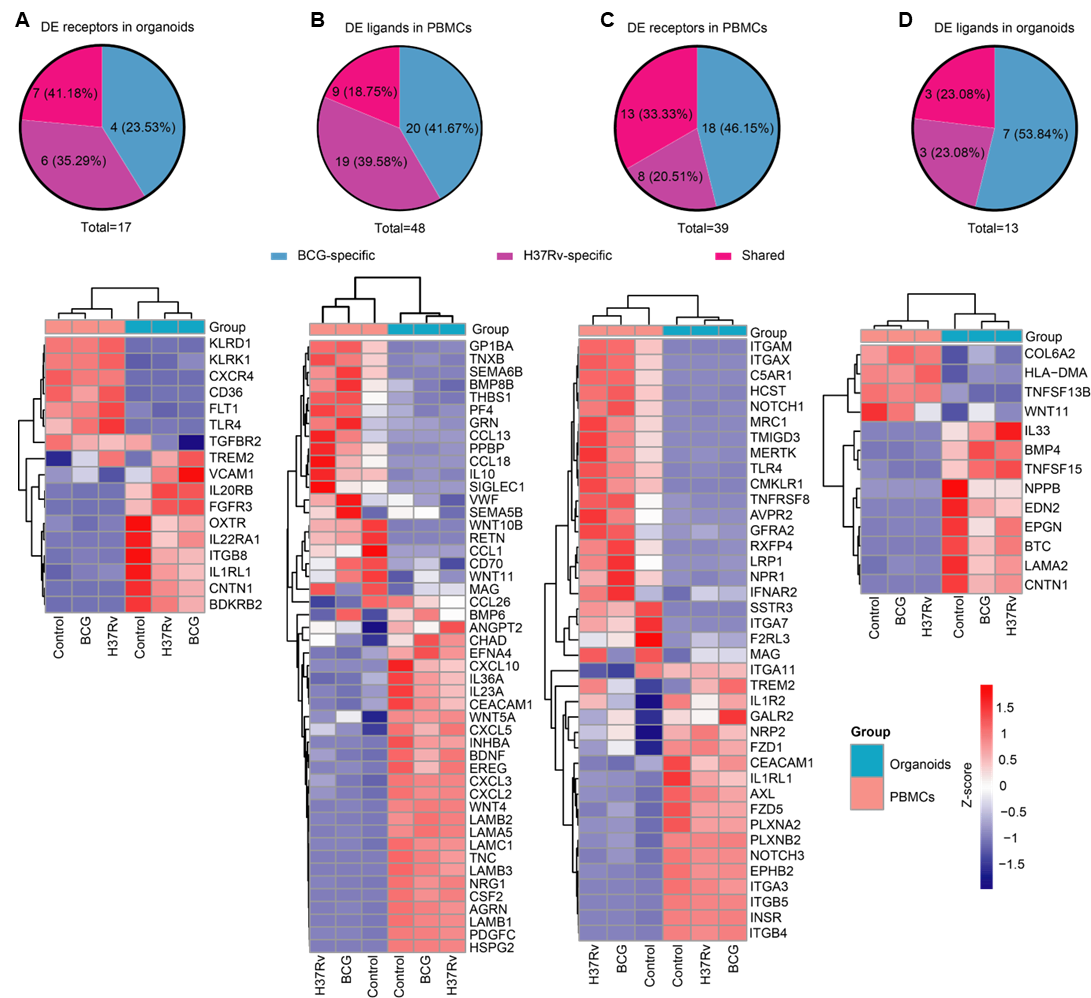
**

**Supplementary figure 9.** DE ligands and receptors in PBMCs and organoids. (A and C) Pie charts displaying the DE receptors specifically regulated by BCG and H37Rv in organoids (A) and PBMCs (C), respectively. The expression patterns of these receptors were included in the following panel. (B and D) Pie charts displaying the DE ligands specifically regulated by BCG and H37Rv in PBMCs (A) and organoids (C), respectively. The expression patterns of these ligands were included in the following panel.

Table S1 Primers for RT-qPCR

| Gene symbol | Forward | Reverse |
| --- | --- | --- |
| *BDNF* | CTACGAGACCAAGTGCAATCC | AATCGCCAGCCAATTCTCTTT |
| *TNFSF15* | GCACCTCTTAGAGCAGACGG | CGGAATGTGACCTGGGAGTAAAT |
| *TNFα* | CCTCTCTCTAATCAGCCCTCTG | GAGGACCTGGGAGTAGATGAG |
| *IL1β* | AGCTACGAATCTCCGACCAC | CGTTATCCCATGTGTCGAAGAA |
| *IL6* | CATCCTCGACGGCATCTCAG | TCACCAGGCAAGTCTCCTCA |
| *IL18* | GTCTCCCAGTGCATTTTGCC | GCAGCCATCTTTATTCCTGCG |
| *HES1* | AAAAATTCCTCGTCCCCGGT | ATGCCGCGAGCTATCTTTCT |
| *HES5* | AGAGAAAAACCGACTGCGGA | GACGAAGGCTTTGCTGTGC |
| *HEY1* | CGGACGAGAATGGAAACTTGA | TGCTCCATTACCTGCTTCTCA |
| *CYR61* | CTCGCCTTAGTCGTCACCC | CGCCGAAGTTGCATTCCAG |
| *CCL2* | CAGCCAGATGCAATCAATGCC | TGGAATCCTGAACCCACTTCT |
| *CTGF* | ACCGACTGGAAGACACGTTTG | TGGAATCCTGAACCCACTTCT |
| *TNFAIP3* | TCCTCAGGCTTTGTATTTGAGC | TGTGTATCGGTGCATGGTTTTA |
| *IFNβ* | GCTTGGATTCCTACAAAGAAGCA | ATAGATGGTCAATGCGGCGTC |
| *GAPDH* | GGAGCGAGATCCCTCCAAAAT | GGCTGTTGTCATACTTCTCATGG |
